# Use of a Smartphone App to Explore Potential Underuse of Prophylactic Aspirin for Preeclampsia

**DOI:** 10.1001/jamanetworkopen.2021.30804

**Published:** 2021-10-29

**Authors:** Tamar Krishnamurti, Alexander L. Davis, Samantha Rodriguez, Laila Hayani, Miriam Bernard, Hyagriv N. Simhan

**Affiliations:** 1 Division of General Internal Medicine, University of Pittsburgh, Pittsburgh, Pennsylvania; 2 Center for Women’s Health Research and Innovation, University of Pittsburgh, Pittsburgh, Pennsylvania; 3 Department of Engineering and Public Policy, Carnegie Mellon University, Pittsburgh, Pennsylvania; 4 Naima Health LLC, Pittsburgh, Pennsylvania; 5 Department of OB-GYN and Reproductive Sciences, University of Pittsburgh, Pittsburgh, Pennsylvania

## Abstract

**Importance:**

Preeclampsia is a leading preventable cause of maternal morbidity and mortality. Initiation of low-dose aspirin (LDASA) treatment at or before 16 weeks’ gestation may prevent preeclampsia onset for patients with specific risk factors.

**Objective:**

To assess potential underuse of LDASA and reasons for underuse using data from a prenatal care smartphone app.

**Design, Setting, and Participants:**

In this prospective cohort study, English-speaking pregnant patients aged 18 years or older from the UPMC health care system received an invitation to use the MyHealthyPregnancy app at their first prenatal appointment. Use of the app was voluntary. The study took place between September 23, 2019, and August 31, 2020, as part of a quality-improvement initiative.

**Exposures:**

The app offered educational information, monitoring tools, and routine screenings tailored to patient-entered gestational age and demographic and clinical characteristics. App-based questions included LDASA eligibility based on US Preventive Services Task Force criteria for preeclampsia risk and a monthly prompt about LDASA recommendations from the patient’s health care practitioner.

**Main Outcomes and Measures:**

The primary outcomes were the receipt of LDASA recommendations from a practitioner and adherence to any such recommendation, as self-reported on the app. Patients’ medical records were examined to cross-reference their self-reports of an LDASA recommendation. Multivariable logistic regression was used to model patient-perceived recommendation as a function of factors associated with preeclampsia.

**Results:**

The patient cohort consisted of 2563 patient participants (2036 [79%] White; mean [SD] age, 30 [5.2] years) with 2567 pregnancies; 1882 pregnancies (73.3%) were among women with private or employer-based insurance, and 1246 (48.5%) were among nulliparous patients. At least 1 factor associated with high risk for preeclampsia was reported in 316 pregnancies (12.3%), and 2 or more factors associated with moderate risk were reported in 1051 (40.9%). Of the 1015 pregnancies for which patients answered voluntary questions about aspirin use, 124 (12.2%) met at least 1 criterion for highest risk of preeclampsia. In 57 (46.0%) of these pregnancies, the patient indicated that their practitioner recommended LDASA; after examination of the medical records, 90 pregnancies (72.6%) had evidence of an LDASA recommendation and 34 (27.4%) did not. Of the 90 pregnancies with a documented LDASA recommendation, 33 patients (36.7%) were unaware of it. Prior preeclampsia (28 weeks’ gestation: odds ratio, 20.1; 95% CI, 11.0-36.9) and chronic hypertension (28 weeks’ gestation: odds ratio, 17.4; 95% CI, 6.3-48.2) were the primary high-risk factors associated with recommendation of LDASA.

**Conclusions and Relevance:**

In this cohort study, only 46.0% of prenatal care app users who met the criteria for highest preeclampsia risk reported receiving an LDASA recommendation from their practitioner, and medical records suggested that there may have been frequent miscommunication between patients and practitioners about LDASA use. Digital tools such as the MyHealthyPregnancy app might offer an opportunity to improve identification of patients at risk for preeclampsia and communication with these patients about aspirin use.

## Key Points

**Question:**

Can potential underuse of prophylactic low-dose aspirin (LDASA) and reasons for underuse be ascertained using data from a prenatal care smartphone app in a quality-improvement setting?

**Findings:**

In this cohort study of 2567 pregnancies among 2563 women in a single health care system, with use of combined data from a prenatal care app and patients’ health records, 27% of patients at high risk for preeclampsia had no LDASA recommendation documented in their medical record. Of the patients with a documented LDASA recommendation, 37% were unaware of it.

**Meaning:**

The findings suggest a need for improvement in identification of patients at risk for preeclampsia and in communication between patients and practitioners about the use of LDASA.

## Teaser

This cohort study compares self-reported data collected from a prenatal care smartphone app with data from pregnant patients’ medical records to assess potential underuse of low-dose aspirin among patients at risk for preeclampsia and reasons for underuse.

## Introduction

Preeclampsia, characterized by new onset of hypertension and proteinuria after 20 weeks’ gestation, is a leading factor associated with maternal and neonatal morbidity and mortality^1^ and occurs in 2% to 8% of all pregnancies.^2^ It is one of the few preventable causes of prematurity, yet it currently is associated with approximately 15% of premature births.^3^ Randomized clinical trials support the use of low-dose aspirin (LDASA) (75-150 mg daily) during pregnancy as an effective prophylactic approach.^4^ However, the association of LDASA with a protective effect has been reported to depend on 3 conditions. The first is appropriate and early identification of patients at moderate to high risk of preeclampsia; prophylaxis has been reported to be optimal if started before 16 weeks’ gestation and may still be associated with improved outcomes if initiated before 28 weeks.^5,6,7^ The second is communication between patients and their prenatal care practitioner, with the receipt of clear communications regarding risk being positively associated with LDASA use.^8^ The third is daily aspirin adherence on the part of the pregnant person,^9^ which review studies have shown may be challenging to achieve.^10,11^ Thus, achievement of the potential benefits of LDASA in a real-world clinical setting would require accurate and quick identification of patients at risk for preeclampsia, clear communication of a prophylactic recommendation to patients, and ensuring adherence to that recommendation.

Comprehensive predictive models for preeclampsia may combine risk factors determined by maternal medical history with physiological measures (eg, blood pressure) and biomarkers (eg, serum placental growth factor).^12,13^ However, maternal medical history is the simplest and most common form of risk assessment, particularly because more detailed protocols require more intensive measurements, including arterial pressure, uterine artery pulsatility index, and biomarkers for placental growth factor.^12^ The US Preventive Services Task Force (USPSTF),^14^ similarly to other national^15^ and international organizations,^16^ has documented a set of criteria suggesting high risk for preeclampsia that warrant LDASA use as a preventive medication, and those criteria have been endorsed by professional societies.^17,18^ In the same issued recommendation, the USPSTF also outlined a set of criteria suggesting moderate risk for which LDASA use should be considered among patients experiencing at least 2 of these criteria. The high-risk criteria include 1 or more of the following: history of preeclampsia, chronic hypertension, type 1 or 2 diabetes, kidney disease, or autoimmune disease such as systemic lupus erythematous or antiphospholipid syndrome. The moderate-risk criteria include 2 or more of the following: obesity (body mass index ≥30) (calculated as weight in kilograms divided by height in meters squared), family history of preeclampsia, nulliparity, sociodemographic characteristics (identifying as Black or African American and/or having low socioeconomic status), advanced maternal age (35 years or older), and history of adverse pregnancy events (specifically, low birth weight or small for gestational age, a previous adverse pregnancy outcome, and/or an interpregnancy interval of more than 10 years). When the comprehensive list of USPSTF clinical risk factors has been used, the detection rate for preeclampsia has been 80% to 90%.^12^

Even with established risk criteria, it may be challenging for practitioners to incorporate a comprehensive baseline risk assessment into routine prenatal care. As a result, individuals at risk of preeclampsia may miss the opportunity to be prescribed an early prophylactic aspirin regimen. Even if at-risk patients are correctly identified based on baseline risk factors and appropriately prescribed an LDASA regimen, tracking their understanding of and adherence to an LDASA regimen may be challenging. Traditional tools for monitoring adherence in trials (eg, metabolite analysis, electronic bottle caps, or ecological momentary assessment) can be costly and burdensome^19^ and thus potentially impractical to implement in routine care.

In this cohort study, we assessed prophylactic aspirin eligibility, comprehension, and use through a mobile health platform. Using data collected through both a prenatal care smartphone app recommended to patients and examination of patients’ electronic medical records, we assessed the 3 required elements of successful LDASA prophylaxis. First, we examined whether individuals meeting the USPSTF high-risk eligibility criteria for aspirin were prescribed LDASA. Then we investigated whether those at high risk of preeclampsia reported an understanding of their LDASA eligibility as explained by their practitioner. Third, we explored whether patients who recalled a recommendation of LDASA from their practitioner reported being adherent to that recommendation. This approach allowed us to assess whether underlying causes of prophylactic LDASA underuse may be detected and examined using data from a prenatal care smartphone app in a quality-improvement setting.

## Methods

In this cohort study, practitioners at UPMC, a large academic health system, recommended the MyHealthyPregnancy (MHP) smartphone app (Apple version 1.4.7 or Android version 1.8)^20^ to adult pregnant patients at their first prenatal appointment. App users consented to share identifiable data with their health care practitioner and for research purposes through 2 distinct app-based electronic consent agreements. As part of the consent, they agreed to the dissemination of anonymized aggregate data for scientific development. Participants did not receive financial compensation for app use. All app use was considered part of routine prenatal care approved by the UPMC health care system’s quality improvement review board as a quality improvement initiative before data collection started. This study followed the Strengthening the Reporting of Observational Studies in Epidemiology (STROBE) reporting guideline.

### Description of the MHP App

The MHP app has several features including educational content tailored to the gestational week, demographic and clinical characteristics of the app user, a fetal movement counter and contraction timer, opportunities to document the pregnancy experience, and routine screenings for symptoms and psychosocial risks. The app offers relevant resources (eg, connection to local health services) or actions (prompts to call 911 or the prenatal care practitioner, instructions for watchful waiting, and prompts to notify the practitioner if critical health risks are documented by the app user) tailored to the information that is entered into the app. The app applies machine learning algorithms to patient-entered data to model an individual patient’s likelihood of experiencing adverse pregnancy events (eg, preeclampsia, depression, or premature delivery). With patient consent and point-of-response reminders about information sharing, select data on specific risk factors identified through the app (eg, depression or reports of decreased fetal movement) are securely transferred to a portal integrated with Epic medical records software (Epic Systems Corporation) that practitioners can access. Although MHP is commercially available, it was initially designed to be used within the UPMC health care system. All content was developed with and reviewed by a clinical education team employed by the health care system.

The internal protocol for recommending MHP was to send a link to patients’ phones inviting them to use the app as part of their routine prenatal care. Users were able to follow the link to a unique download code in the smartphone app store. During their first use of MHP, all app participants were prompted with multiple-choice questions assessing USPSTF risk criteria for taking low-dose aspirin. Subsequently, MHP prompted users with 2-monthly questions: “Since you’ve been pregnant, has your provider recommended that you take aspirin?” and “Over the last month, how often have you taken aspirin?”

### Statistical Analysis

Data were analyzed from participants who downloaded and initiated use of the MHP app from September 23, 2019, to August 31, 2020. Means (SDs) and counts (percentages) were used to characterize self-reported patient demographic characteristics and clinical factors associated with preeclampsia. Multivariable logistic regression using maximum-likelihood estimation with odds ratios and 95% CIs was used to model patient-perceived aspirin recommendation as a function of USPSTF factors suggesting high risk of preeclampsia. We also report the results of a model based on single factors suggesting moderate risk after controlling for high-risk factors. Regression analyses used data from patients who were invited to use the app, initiated its use, and answered the question about whether their practitioner recommended LDASA. Statistical analyses also used unique pregnancies rather than unique patients. All 95% CIs in the logistic regressions are given with the assumption that the log-odds coefficients were normally distributed.

The USPSTF criteria were elicited before determination of multifetal gestation, and statistical analyses were based on self-reported data collected before 16 weeks’ gestation unless explicitly stated otherwise. Each regression analysis used a complete-cases approach including only the observations that had the complete data required for that particular analysis. Imputation was not used for the dependent or independent variables. All statistical analyses were completed using Python, version 3.8.8 (Python Software Foundation). Statistical significance was set at 2-tailed *P* < .05. Because there was potential for type I errors owing to multiple comparisons and the analysis was not preplanned, the findings of these analyses should be interpreted as exploratory. All the analyses were independently replicated by 2 data analysts (S.R. and L.H.). Any discrepancies were resolved by rerunning the analyses and by discussion.

## Results

### Participants

The MHP app was recommended to 3484 users, and of these, 2563 (73.6%) downloaded the app and agreed to share their data during the study period (Figure). Four of these participants used the app for 2 pregnancies, giving a total of 2567 pregnancies recorded in the app. The mean (SD) age of participants was 30 (5.2) years, and most (2036 [79.3%]) were White. A total of 1740 pregnancies (67.8%) were among patients who had a college degree, 1882 (73.3%) were among patients who had private or employer-based health insurance, and 1246 (48.5%) were among patients experiencing their first pregnancy (Table 1). The MHP app is currently only available at UPMC in English; therefore, patients who did not speak English self-excluded from the study. The median gestational age during which the invitation to use MHP was received was 10 weeks (range, 4-40 weeks). Most patients received the invitation at their first prenatal visit. More detailed information on demographic differences between patients who were or were not invited to use MHP and between those who did or did not initiate use is provided in eTable 1 in the Supplement.

**Figure.**
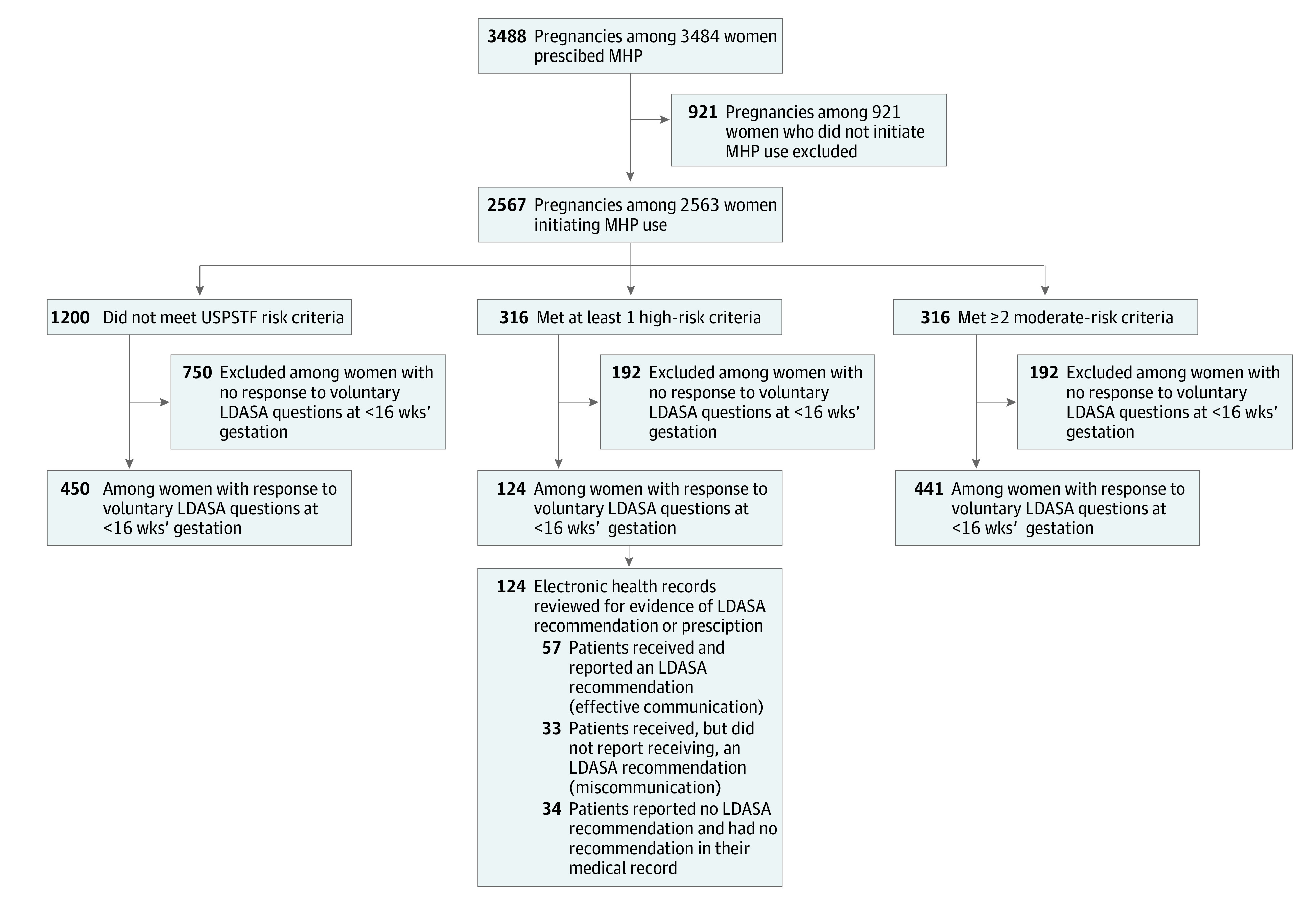
Selection of Study Participants From Among Patients Using the MyHealthyPregnancy (MHP) App The MHP app was recommended to all English-speaking pregnant patients aged 18 years or older. Four of these participants used the app for 2 pregnancies. LDASA indicates low-dose aspirin; USPSTF, US Preventive Services Task Force.

**Table 1.** Demographic Characteristics in Pregnancies Among Patients Using MHP and the Larger UPMC Prenatal Population

Variable	Pregnancies among patients using MHP (N = 2567)^a^	Total clinic population (N = 17 901)^b^
Age, mean (SD), y	30 (5.2)	29 (5.5)
Income, US dollars, thousands		
<15	308 (12.0)	NA
15 to <50	589 (23)	NA
50-100	770 (30.0)	NA
>100	788 (30.7)	NA
Missing or preferred not to respond	112 (4.4)	NA
Race and ethnicity^c^		
Black or African American	267 (10.4)	2737 (15.3)
East Asian	52 (2.0)	185 (1.0)
Hispanic or Latino	49 (1.9)	554 of 26 670 (2.1)
Native American	9 (0.4)	64 (0.4)
South Asian	56 (2.2)	299 (1.7)
White	2036 (79.3)	13 880 (77.5)
Other^d^	79 (3.1)	424 (2.4)
Missing or preferred not to respond	19 (0.7)	312 (1.7)
Educational level		
No high school or GED	66 (2.6)	NA
High school or GED	731 (28.5)	NA
Associate’s degree	303 (11.8)	NA
Bachelor’s degree	726 (28.3)	NA
Postgraduate	711 (27.7)	NA
Missing or preferred not to respond	30 (1.2)	NA
Insurance type^e^		
Private or employer-based	1882 (73.3)	10 706 (59.8)
Medicare or Medicaid	612 (23.8)	6757 (37.7)
Self-pay or other	73 (2.8)	438 (2.4)
Missing	0	0
BMI		
Mean (SD)	27.8 (7.4)	27.9 (7.3)
Missing or preferred not to respond	6 (0.2)	2104 (11.8)
Parity		
Nulliparous	1246 (48.5)	7782 (43.5)
Multiparous	1321 (51.5)	9702 (54.2)
Missing	0	417 (2.3)
Multifetal gestation		
Singleton	2546 (99.2)	17 404 (97.2)
Multiples	21 (0.8)	363 (2.0)
Missing	0 (0)	134 (0.7)

Abbreviations: BMI, body mass index (calculated as weight in kilograms divided by height in meters squared); GED, General Educational Development Test; MHP, MyHealthyPregnancy smartphone application; NA, not available.

^a^ Data are presented as the number (percentage) of patients unless otherwise indicated.

^b^ Clinic population values were determined by aggregate UPMC data. Some demographic data are not collected or stored by UPMC. Data are presented as the number (percentage) of patients.

^c^ UPMC population percentages sum to more than 100% because UPMC records ethnicity and race separately, whereas the MHP app records them in the same question.

^d^ Other included Filipino, Guam or Chamorro, other Asian, other Pacific Islander, and Vietnamese.

^e^ Insurance type was not self-reported and was extracted from the medical records after patient deliveries.

### Distribution of Factors Associated With Preeclampsia

All participants responded to the high-risk criteria questions. A total of 316 of the app users (12.3%) met at least 1 of the USPSTF high-risk criteria. Participants who reported 2 or more moderate-risk criteria but no high-risk factors constituted 40.9% of the patient population (1051 patients). The distribution of risk criteria in the sample is shown in Table 2.

**Table 2.** Distribution of Preeclampsia Risk Criteria Among All Pregnancies

Variable	Pregnancies, No. (%) (N = 2567)
High risk	
Chronic hypertension	68 (2.6)
Prior preeclampsia	137 (5.3)
Type 1 or 2 diabetes	47 (1.8)
Autoimmune disease	96 (3.7)
Kidney disease	4 (0.2)
Moderate risk	
Prior adverse outcomes^a^	738 (28.7)
Obesity^b^	776 (30.2)
Maternal age ≥35 y	438 (17.1)
Nulliparity	1246 (48.5)
Identifying as Black or African American	267 (10.4)
Family history of preeclampsia	191 (7.4)
Annual household income <$25 000	495 (19.3)

^a^ For example, previous preterm infant.

^b^ Body mass index (calculated as weight in kilograms divided by height in meters squared) of 30 or greater.

### Association of Risk Factors With Practitioner Recommendation of LDASA

Table 3 shows the high-risk and moderate-risk criteria associated with patient reports of receiving an LDASA recommendation from their practitioner at 2 time cutoffs. The first was at or before 16 weeks’ gestation, which is the optimal time for initiating prophylactic LDASA.^17^ The second was at or before 28 weeks’ gestation, which is still considered an effective time frame for prophylaxis.^17^ Practitioners included obstetrics and gynecology residents and attending physicians, midwives, nurse practitioners, physician assistants, and maternal-fetal medicine physicians.

**Table 3.** Criteria Associated With Patients’ Odds of Receiving an Aspirin Recommendation, by Gestational Time Frame

Variable	Odds ratio (95% CI)	
	Gestation ≤16 wk	Gestation ≤28 wk
High risk		
Pregnancies, No.	1015	1234
Chronic hypertension	14.1 (5.2-38.3)	17.4 (6.3-48.2)
Prior preeclampsia	12.0 (6.4-22.5)	20.1 (11.0-36.9)
Type 1 or 2 diabetes	5.8 (2.2-15.2)	8.6 (3.6-20.6)
Autoimmune disease	3.9 (1.9-8.0)	3.4 (1.7-6.5)
Kidney disease^a^	0.5 (0.0-6.3)	0.3 (0.0-3.7)
Moderate risk^b^		
Pregnancies, No.	1012	1231
Prior adverse outcomes^c^	3.1 (1.6-6.1)	2.3 (1.3-4.1)
Obesity^d^	2.3 (1.5-3.5)	2.8 (1.9-4.0)
Maternal age ≥35 y	1.8 (1.1-3.0)	2.1 (1.3-3.2)
Nulliparity	2.5 (1.2-5.0)	2.2 (1.3-3.9)
Identifying as Black or African American	1.9 (1.1-3.5)	2.0 (1.2-3.3)
Family history of preeclampsia	0.8 (0.4-1.8)	0.8 (0.4-1.5)
Annual household income<$25 000	0.8 (0.4-1.3)	0.7 (0.5-1.2)

^a^ The number of participants with self-reported kidney disease was small (4), limiting the conclusions that could be drawn from the odds ratio for this variable. However, it was retained in the model for completeness.

^b^ All criteria associated with moderate risk that were included in the regression were controlled for the presence of any high-risk criteria. US Preventive Services Task Force criteria were elicited before determination of multifetal gestation.

^c^ For example, previous preterm infant.

^d^ Body mass index (calculated as weight in kilograms divided by height in meters squared) of 30 or greater.

At both 16 and 28 weeks’ gestation, history of preeclampsia (16 weeks: OR, 12.0 [95% CI, 6.4-22.5]; 28 weeks: OR, 20.1 [95% CI, 11.0-36.9]) and of chronic hypertension (16 weeks: OR, 14.1 [95% CI, 5.2-38.3]; 28 weeks: OR, 17.4 [95% CI, 6.3-48.2]) were the primary high-risk factors associated with practitioner LDASA recommendation. However, the ORs suggested that all risk criteria were being underused by practitioners (ORs should diverge to infinity for all high-risk criteria). After controlling for high-risk criteria, the following moderate-risk criteria were associated with a recommendation for aspirin use at both times (data shown for 28 weeks’ gestation): prior adverse pregnancy outcomes (OR, 2.3; 95% CI, 1.3-4.1), a body mass index of 30 or greater (OR, 2.8; 95% CI, 1.9-4.0), a maternal age of 35 years or older (OR, 2.1; 95% CI, 1.3-3.2), first-time pregnancies (OR, 2.2; 95% CI, 1.3-3.9), and/or identifying as Black or African American (OR, 2.0; 95% CI, 1.2-3.3). Family history of preeclampsia and low annual household income were not associated with LDASA recommendation, counter to USPSTF guidelines. Patients with 2 or more moderate-risk factors but no high-risk factors reported low rates of LDASA prescription, ranging from 3.6% to 6.7% (Table 4).

**Table 4.** Distribution of Pregnancies Among Participants Who Had 2 or More US Preventive Services Task Force Moderate-Risk Factors Associated With Preeclampsia and Were Prescribed Aspirin

Risk factor	Pregnancies prescribed aspirin, No./total No. with factors (%)
Prior adverse outcomes and at least 1 other factor associated with moderate risk	26/389 (6.7)
Obesity and at least 1 other factor associated with moderate risk	37/551 (6.7)
Advanced maternal age and at least 1 other factor associated with moderate risk	21/313 (6.7)
Nulliparity and at least 1 other factor associated with moderate risk	27/560 (4.8)
Identifying as Black or African American and at least 1 other factor associated with moderate risk	14/216 (6.5)
Family history of preeclampsia and at least 1 other factor associated with moderate risk	6/128 (4.7)
Low annual household income and at least 1 other factor associated with moderate risk	14/389 (3.6)

In 1015 pregnancies, the patients answered voluntary questions about aspirin use; in 124 (12.2%) of these, the pregnancy met at least 1 criterion for highest risk of preeclampsia and the pregnant patient responded to the in-app question about an aspirin recommendation from their practitioner before 16 weeks’ gestation. Medical records for these 124 patients were examined. The medication list and medical record notes were reviewed for a documented prescription or written recommendation of over-the-counter LDASA from a prenatal care practitioner. Of these 124 patients, 90 (72.6%) had a documented LDASA recommendation in their medical record, and 34 (27.4%) did not. More details on the proportion of respondents who received aspirin recommendations for each high-risk factor are provided in eTable 2 in the Supplement.

### Patient-Practitioner Communication About Risk of Preeclampsia

Of the 90 patients meeting at least 1 high-risk criterion for preeclampsia who responded to the LDASA questions and had an LDASA recommendation documented in their medical records, 57 (63.3%) reported being aware that their practitioner had recommended LDASA and 33 (36.7%) reported being unaware of receiving an aspirin recommendation. All high-risk participants without a documented LDASA recommendation accurately reported not having received one.

### Patient Self-reported Adherence

Of the 2567 pregnancies in the study, 1552 (60.5%) had missing self-reported LDASA data. A total of 132 patients reported receiving an aspirin recommendation, regardless of risk level or whether it was indicated in their medical record, and of these, 64 (48.5%) said they took aspirin in adherence with guidelines (55 responded “every day,” and 9 responded “several times per week”). Of the 57 patients at high risk for preeclampsia who were aware of the aspirin recommendation documented in their medical record, 27 (47.4%) were adherent with guidelines. A lower likelihood of adherence was found among the 132 patients who reported receiving an aspirin recommendation and responded to the question of aspirin adherence (χ^2^, 4.05; *P* = .04). However, self-reported adherence did not otherwise significantly differ across primary risk factors or patient demographics. Additional data on the distribution of patients reporting changes in aspirin adherence during the first and second trimesters are shown in eTable 3 in the Supplement.

## Discussion

### Principal Findings

Low-dose aspirin, if prescribed early, is an evidence-based strategy for reducing the chance of developing preeclampsia among patients at high risk.^14^ Adherence with this treatment has been associated with improved outcomes.^21^ In this cohort study, we used a prenatal care app to solicit USPSTF preeclampsia risk criteria from patients early in the first trimester. Less than half of patients (46.0%) who were identified as eligible for LDASA according to their high-risk criteria reported that they had received an aspirin recommendation from their practitioner by 16 weeks’ gestation. Patients’ medical records confirmed that in 27.4% of pregnancies meeting high-risk criteria for preeclampsia, the pregnant patients had no mention of LDASA in their medication list or clinical notes. For patients at high risk of preeclampsia who had an LDASA recommendation documented in their medical record, 36.7% were unaware of it, suggesting gaps in communication about LDASA eligibility. Overall, these findings suggest an opportunity for better baseline risk identification and a need for clear patient-practitioner communication about aspirin and preeclampsia risk.

The findings also suggest that some risk factors were more commonly used than others when practitioners decided to prescribe LDASA for prenatal patients. Moreover, in the absence of a high-risk factor, the presence of multiple moderate-risk factors was rarely associated with an LDASA recommendation, despite USPSTF guidelines. The practitioners’ greater reliance on certain risk factors, such as history of preeclampsia and chronic hypertension, may have been attributable to the perceived strength of the predictive value of these factors. Lesser use of other criteria, such as autoimmune disease, may have been the result of some risk factors being out of the scope of routine prenatal care and more frequently managed by maternal-fetal medicine or other specialists such as rheumatologists. Use of certain risk factors incorrectly or not at all may have resulted from lack of information about whether the patient met the risk criteria (eg, the patient’s annual household income) or a lack of time available to elicit certain maternal factors (eg, family history) or may indicate that patients with specific risk criteria may also experience greater barriers to accessing preventive care. Underuse or inaccurate use of factors not only may lead to underestimates of overall preeclampsia risk but may also exacerbate disparities in preventive preeclampsia care, such as among those with low socioeconomic status.

### Clinical Implications

In 2017, a systematic review^22^ was published of the evidence informing the USPSTF preeclampsia recommendation.^14^ That review^22^ concluded that evidence of benefits and harms was limited for the existing screening protocols for preeclampsia risk. In addition to an assessment of baseline medical history, preeclampsia risk screenings often include point-of-care urine tests and repeated blood pressure measurements.^23^ Other risk models not yet incorporated into routine clinical practice include biomarker levels obtained from blood samples.^14^ Such physical risk assessments may be invasive or unduly worrisome to patients^24^ and have mixed predictive value.^12^ In this study, patients were willing to share baseline risk factors through a mobile health app and had few missing data on those risk factors, suggesting that this approach to screening may be less invasive or stressful than other screening methods even if it is less sensitive than other methods such as routine blood pressure monitoring. Because of the low risk associated with LDASA use, an app-based approach to universal screening and LDASA recommendation may offer a helpful way to complete early risk identification and wide-scale intervention.

This study’s findings also provide information on the specific risk factors that practitioners may be more frequently considering and those that may be underused when making clinical judgments. This finding is consistent with broader literature discussing physician use of heuristics for diagnosis and medication prescription.^25^ Of the patients in this study who reported that their practitioner had recommended aspirin, 48.5% reported a high level of adherence to that recommendation. However, 36.7% of patients with a documented LDASA recommendation in their medical record were unaware of that recommendation. LDASA for preeclampsia risk is the standard of care in the UPMC health care system. It is possible that some practitioners using an electronic medical record template added an LDASA recommendation to patients’ medical records without ever engaging in a conversation with the patients about their LDASA eligibility. It can be challenging for practitioners to adequately communicate risk information to patients, particularly regarding complex, uncertain, or unfamiliar issues.^26,27^ Moreover, pregnant patients may feel wary of medication use, especially when the benefits are unclear to them. As a result, even if a conversation with a patient about LDASA eligibility occurs, it is possible that the conversation and any plan-of-care materials offered may not provide the information in a way that allows the patient to make a fully informed decision.^28^ Data from a prenatal app may offer an opportunity to identify patients requiring better communication from their health care practitioner about their eligibility for prophylactic aspirin, and electronic best-practice alerts embedded in the health record may offer an opportunity to prompt practitioners to incorporate risk factors that are underused.

Given the minimal risks of low-dose aspirin, there are some proponents of universal LDASA use, which could make new approaches to screening less applicable for risk identification or practitioner decision-making.^29^ This study’s findings, however, suggest that digital tools may offer an opportunity not just for screening but also for identifying patient-practitioner miscommunications about LDASA benefits. If universal screening is implemented, engagement with patient-facing digital tools may be beneficial in communicating how underlying risk can be addressed with LDASA, ultimately increasing patient uptake and adherence.^30^

### Limitations

This study has limitations. The analyses were performed only for patients who downloaded and consented to use the app. This represented a modest proportion of the larger prenatal care patient population. Moreover, among those who consented to use the app, reporting of all data was voluntary, and as such, self-reported LDASA data were missing for 60.5% of the patients in the study sample. Missing data are not unusual in real-world settings (eTable 5 in the Supplement shows no demographic differences between patients in this study who chose to respond to questions and those who chose not to respond). However, missingness may have introduced selection bias if those who chose to not respond to the questions about aspirin recommendation had a different degree of understanding of their practitioner’s recommendation than did those who chose to respond regardless of demographic characteristics. Whereas there were almost no missing data on risk criteria, self-reports leave room for error in recall with respect to LDASA recommendation and use (levels of consistency between self-report and electronic health record data are show in eTable 4 in the Supplement). Even data obtained from the patients’ electronic health records may not have fully captured informal conversations between patients and practitioners. As such, it is unclear whether the absence of an LDASA recommendation by a practitioner reflected a true documented absence or poor documentation of a conversation-based recommendation.

It is possible that when making aspirin recommendations, practitioners were integrating information not documented through the app or in patients’ medical records. For example, MHP asked about autoimmune disease as a USPSTF criteria. However, not all autoimmune diseases are associated with equivalent risk of preeclampsia or meet the criteria for low-dose aspirin (eg, thyroid disease).^31^ More information is needed on how prenatal health care practitioners make risk distinctions when incorporating autoimmune disease diagnoses into their clinical judgments about LDASA eligibility. It is also possible that other measures of preeclampsia risk, such as routine blood pressure monitoring, have a greater role in physician decision-making than does maternal medical history. However, as telemedicine and distance monitoring become more routine and acceptable means of administering prenatal care,^32,33^ simple risk-prediction tools embedded in digital health apps show promise for identifying instances of potential preeclampsia risk that would otherwise be missed. Moreover, such tools offer an opportunity to improve and refine simple risk-prediction models. Paired with blood pressure cuffs, for example, they could offer an opportunity for even earlier and more robust risk prediction.

## Conclusions

In this cohort study, through the use of patient-entered data in a smartphone app, we revealed underuse of prophylactic LDASA by pregnant patients. The findings suggest that there may be a gap in communication between patients and practitioners about preeclampsia risk and aspirin eligibility. In addition, the findings suggest that low levels of prophylactic aspirin use among pregnant women at risk for preeclampsia may be in part attributable to incomplete use of risk factors by prenatal care practitioners. Overall, the findings suggest that mobile health tools have the potential to offer an opportunity for low-burden, early identification of pregnant patients who would benefit from prophylactic aspirin as well as those who do not adhere to the aspirin regimen, particularly if that behavior is associated with poor communication between the patient and practitioner. Future work should examine the potential of smartphone apps to support communication between patients and practitioners and increase understanding of LDASA use among patients identified as having risk for preeclampsia.

## Notes

Supplement. **eTable 1.** Results of a multivariable logistic regression of prenatal app prescription and initiation **eTable 2.** Distribution of high-risk factors, LDASA response, and adherence **eTable 3.** Changes in self-reported aspirin adherence **eTable 4.** Instances of data discrepancies between available self-reported and electronic health record data for all study participants (N = 1702) **eTable 5.** Relationship between demographic factors and likelihood of LDASA response
