# Supplementary material for: Use of a Smartphone App to Explore Potential Underuse of Prophylactic Aspirin for Preeclampsia
Source: JAMA Netw Open. 2021 Oct 29;4(10):e2130804. doi: 10.1001/jamanetworkopen.2021.30804 (PMC8556626; doi:10.1001/jamanetworkopen.2021.30804)
Supplement: Supplement. — eTable 1. Results of a multivariable logistic regression of prenatal app prescription and initiation eTable 2. Distribution of high-risk factors, LDASA response, and adherence eTable 3. Changes in self-reported aspirin adherence eTable 4. Instances of data discrepancies between available self-reported and electronic health record data for all study participants (N = 1702) eTable 5. Relationship between demographic factors and likelihood of LDASA response [file jamanetwopen-e2130804-s001.pdf]

Supplementary Online Content

Krishnamurti T, Davis AL, Rodriguez S, Hayani L, Bernard M, Simhan HN. Use of a smartphone app to explore potential underuse of prophylactic aspirin for preeclampsia. *JAMA Netw Open*. 2021;4(10):e2130804. doi:10.1001/jamanetworkopen.2021.30804

eTable 1. Results of a multivariable logistic regression of prenatal app prescription and initiation
eTable 2. Distribution of high-risk factors, LDASA response, and adherence
eTable 3. Changes in self-reported aspirin adherence
eTable 4. Instances of data discrepancies between available self-reported and electronic health record data for all study participants (N = 1702)
eTable 5. Relationship between demographic factors and likelihood of LDASA response

This supplementary material has been provided by the authors to give readers additional information about their work.

© 2021 Krishnamurti T et al. *JAMA Network Open*.

Supplemental Material

eTable 1. Results of a multivariable logistic regression of prenatal app prescription and initiation

**Demographic or Risk Factor**	**Odds Ratio [Confidence Interval] of app prescription (n=17901)**	**Odds Ratio [Confidence Interval] of initiating app use (n=2193)**
Race	1.01 [1.00, 1.03]	1.02 [1.00, 1.06]
Insurance Type	0.60 [0.55, 0.66]	0.66 [0.52, 0.82]
Advanced Maternal Age	0.91 [0.80, 1.04]	1.33 [0.93, 1.92]
Obese Body Mass Index	1.08 [0.97, 1.20]	1.27 [0.96, 1.67]
Nulliparity	1.58 [1.43, 1.75]	1.18 [0.91, 1.52]
Chronic Hypertension	0.89 [0.67, 1.19]	1.23 [0.55, 2.75]
Prior Preeclampsia	1.09 [0.84, 1.42]	1.35 [0.65, 2.79]
Type I or II Diabetes	1.00 [0.59, 1.67]	1.34 [0.30, 6.01]
Kidney Disease	1.00 [1.00, 1.00]	1.00 [1.00, 1.00]

© 2021 Krishnamurti T et al. *JAMA Network Open*.

eTable 2. Distribution of high-risk factors, LDASA response, and adherence

**Risk Factor History:**	**Chronic Hypertension**	**Prior Preeclampsia**	**Type I or Type II Diabetes**	**Autoimmune Disease**	**Kidney Disease**
Frequency (Percent) among high-risk patients <16 weeks' gestation	68/316 (21.5%)	137/316 (43.4%)	47/316 (14.9%)	96/316 (30.4%)	4/316 (1.3%)
Frequency (Percent) who responded to Aspirin Questions	22/68 (32.4%)	50/137 (36.5%)	22/47 (46.8%)	43/96 (44.8%)	3/4 (75.0%)
Frequency (Percent) who reported a positive Aspirin Recommendation	15/22 (68.2%)	29/50 (58.0%)	10/22 (45.5%)	14/43 (32.6%)	1/3 (33.3%)
Frequency (Percent) with Aspirin Adherence	6/15 (40.0%)	15/29 (51.7%)	6/10 (60.0%)	8/14 (57.1%)	0/1 (0.0%)

© 2021 Krishnamurti T et al. *JAMA Network Open*.

eTable 3. Changes in self-reported aspirin adherence

**Aspirin Adherence among those with an aspiring prescription/recommendation**	**Frequency (Percent) for <16 weeks**	**Frequency (Percent) for <28 weeks**
Always adherent	60/132 (45.5%)	103/187 (55.1%)
Always non-adherent	39/132 (29.5%)	47/187 (25.1%)
Report adherence and non-adherence	33/132 (25.0%)	37/187 (19.8%)
Majority (>50%) of responses report adherence	82/132 (62.1%)	128/187 (68.4%)
Minority (≤50%) of responses report adherence	50/132 (37.9%)	59/187 (31.6%)

© 2021 Krishnamurti T et al. *JAMA Network Open*.

eTable 4. Instances of data discrepancies between available self-reported and electronic health record data for all study participants (n=1702)

**Variable**	**Discrepant data**
	Frequency (Percent)
Race/Ethnicity	133/1702 (7.8%)
Chronic Hypertension	52/1702 (3.1%)
Prior Preeclampsia	84/1702 (4.9%)
Type I or II Diabetes	20/1702 (1.2%)
Kidney Disease	3/1702 (0.2%)

© 2021 Krishnamurti T et al. *JAMA Network Open*.

eTable 5. Relationship between demographic factors and likelihood of LDASA response

Demographic Factors	Odds Ratio [Confidence Interval] for 16 Weeks	Odds Ratio [Confidence Interval] for 28 Weeks
Age	0.99 [0.97, 1.00]	0.98 [0.96, 0.99]
Income	0.88 [0.83, 0.94]	0.91 [0.85, 0.97]
Race/Ethnicity	0.99 [0.95, 1.02]	0.97 [0.93, 1.00]
Education	0.96 [0.91, 1.02]	0.98 [0.93, 1.04]
Insurance	1.12 [0.96, 1.32]	1.17 [1.00, 1.37]

© 2021 Krishnamurti T et al. *JAMA Network Open*.
